# Study on the effects of polymer modifiers and phloem girdling on cotton in cadmium-contaminated soil in Xinjiang Province, China

**DOI:** 10.1038/s41598-020-63421-w

**Published:** 2020-04-14

**Authors:** MengJie An, Changzhou Wei, Kaiyong Wang, Hua Fan, Xiaoli Wang

**Affiliations:** Agriculture College, Shihezi University, Shihezi, Xinjiang 832003 China

**Keywords:** Plant physiology, Wounding

## Abstract

The effects of two liquid modifiers (polyacrylate compound modifier and organic polymer compound modifier) and phloem girdling (stem girdling and branch girdling) on cadmium (Cd) content, Cd transport, and photosynthetic parameters of cotton (Xinluzao 60) in Cd-contaminated soil (40 mg kg ^−1^) were studied through barrel experiment. The results showed that the distribution ratios of Cd in stem, leaves, and bolls, leaf net photosynthetic rate (*Pn*), leaf stomatal conductance (*Gs*), leaf transpiration rate (*Tr*), and chlorophyll content were decreased after girdling; and the application of modifiers reduced the Cd content and the Cd transported to the shoot, while alleviating photosynthetic damage caused by girdling. In general, our results indicated that the inhibition of carbohydrate supply caused by girdling reduced the photosynthetic capacity of cotton, while the applications of the two liquid modifiers decrease the influence to cotton photosynthesis. Moreover, Cd and modifiers may be transported to the shoot through both phloem and xylem.

## Introduction

Cadmium (Cd) is a toxic heavy metal commonly found in agricultural soils^1^. It is not necessary for plants, but it is easily absorbed by plant root and enriched in different tissues and organs^2^. Therefore, many scholars have studied the accumulation of Cd in crops and tried to reduce the uptake of Cd by crops. For example, Sebastian *et al*.^3^ found that the application of organic acids reduced the accumulation of Cd in plant leaves, while the application of malic acid reduced the accumulation of Cd in plant roots. Shi *et al*.^4^ found that super absorbent polymer (SAP) immobilized heavy metals in soil, which had a positive effect on maize photosynthesis and growth. Moreover, to reduce the accumulation of Cd, it is necessary to understand the mechanism of Cd transport in plants^5,6^. Mori *et al*.^7^ indicated that the xylem unloading limited the transport of Cd from root to shoots of wild eggplant. Qin *et al*.^8^ found that phloem played a leading role in the transport of Cd from root to leaves. However, there is no conclusive study on the transport of Cd in cotton at present.

Photosynthesis is a biological process in which plants convert light energy into chemical energy that can be used in life processes and synthesize organic matter. It is the physiological basis of plant survival and one of the most basic physiological processes in plant production^9^. However, Cd may inhibit photosynthesis of plants^10^. Moradi and Ehsanzadeh^11^ reported that Cd inhibited chlorophyll synthesis, resulting in a decrease in the amount of light-harvesting chlorophyll a/b-binding protein. Paunov *et al*.^12^ reported that Cd interfered with the photosynthetic electron-transfer process and decreased the efficiency of energy conversion in photosystem II. Therefore, the remediation of Cd pollution has been studied through plant photosynthesis regulation. For example, Liu *et al*.^13^ found that the application of salicylic acid significantly reduced Cd absorption in most Cd-stressed plants and restored photosynthetic efficiency. An *et al*.^14^ found that the application of liquid modifiers to Cd-contaminated soil increased the gas exchange and photosynthetic pigment content of plant leaves.

Cotton (*Gossypium hirsutum*) is one of the main economic crops in the arid region of northern China, and drip irrigation has been widely used in cotton cultivation. Drip irrigation has obvious water-saving and yield-increasing features. In addition, it can be combined with fertilization to obviously increase the fertilizer efficiency^15^. Therefore, whether the application of modifiers through drip irrigation also has an obvious effect on the remediation of soil heavy metal pollution is worth exploring.

At present, most researches on the remediation of soil heavy metal pollution focus on conventional solid materials, but conventional solid materials are not suitable for drip irrigation systems; and the transport and accumulation of Cd in cotton organs have not been determined. Therefore, the study of how liquid polymer modifiers affect cotton in Cd-contaminated soils has important implications for avoiding the potential risks of heavy metals in arid regions. In this study, the effects of two liquid modifiers (polyacrylate compound modifier and organic polymer compound modifier) and phloem girdling (stem girdling and branch girdling) on Cd content, Cd transport, and photosynthetic parameters of cotton (Xinluzao 60) in Cd-contaminated soil (40 mg kg^−1^) were studied through barrel experiment. This study aimed to clarify the distribution and transformation of Cd in cotton, and to explore the remediable mechanism of modifiers. We hypothesized that: (1) the transport pathways of Cd and modifiers in cotton were similar; (2) the modifiers affected the transport and accumulation of Cd in cotton organs; (3) the modifiers changed the photosynthetic characteristics of cotton leaves.

## Results

### Effects of girdling and modifier application on Cd content in different organs of cotton

**Figure 1:**
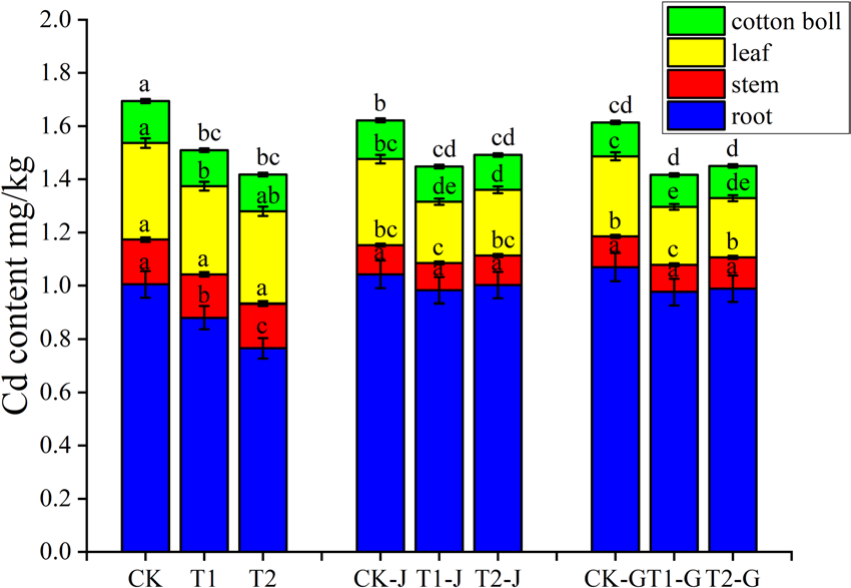
Cadmium contents in different organs of cotton under different treatments. Means in each group followed by the same lowercase letters are not significantly different (*P* < 0.05) by Duncan’s multiple range test. Data are means ± SD (n = 3).

The root of cotton had the highest Cd content, followed by leaves, stem, and cotton bolls (Fig. 1). After the application of the two modifiers, the contents of Cd in root, stem, leaves, and bolls were decreased compared with CK; among them, the decreases of Cd contents in root and bolls were significant. Compared with CK, the Cd contents in root for T1 and T2 groups decreased by 12.33% (*P* < 0.05) and 23.79% (*P* < 0.05), respectively, whereas those in bolls for T1 and T2 groups decreased by 14.48% (*P* < 0.05) and 12.87% (*P* < 0.05), respectively. Compared with CK, the Cd contents in root for CK-J and CK-G groups increased by 3.76% and 6.48%, respectively, whereas the Cd contents in stem, leaves, and bolls all decreased (*P* < 0.05). Compared with CK-J group, the Cd contents in root, leaves, and bolls for T1-J group decreased by 5.71%, 28.84% (*P* < 0.05), and 8.75% (*P* < 0.05), respectively, and those for T2-J group decreased by 3.87%, 23.59% (*P* < 0.05) and 9.9% (*P* < 0.05), respectively. Compared with CK-G group, the Cd contents in root, leaves, and bolls for T1-G group decreased by 8.73%, 27.63% (*P* < 0.05), and 5.98%, respectively, and those for T2-J group decreased by 7.61%, 26.11% (*P* < 0.05), and 5.00%, respectively.

### Effects of girdling and modifiers on cadmium transport

The distribution ratio and transport index of Cd in different organs of cotton under different treatments were shown in Table 1. Compared with CK, the distribution ratios of Cd in root and bolls for T1 group decreased by 1.55% and 3.97%, respectively, whereas those in stem and leaves increased by 7.68% (*P* < 0.05) and 2.45%, respectively; the distribution ratio of Cd in root for T2 group decreased by 8.91%, whereas those in stem, leaves, and bolls increased by 18.95% (*P* < 0.05), 14.07% (*P* < 0.05), and 4.14%, respectively. That is, the application of modifiers reduced the Cd content in root but increased the Cd content in stem, leaves, and bolls. After stem girdling, the distribution ratio of Cd in root for CK-J group increased by 8.43%, whereas those in stem, leaves, and bolls decreased by 31.83% (*P* < 0.05), 6.85%, and 3.91%, respectively, compared with CK. Compared with CK-J group, the distribution ratios of Cd in root, stem, and bolls for T1-J group increased by 5.57%, 4.18%, and 2.16%, respectively, whereas that in leaves decreased by 20.33% (*P* < 0.05); the distribution ratios of Cd in root and stem for T2-J group increased by 4.52% and 9.82%, respectively, whereas those in leaves and bolls decreased by 16.93% (*P* < 0.05) and 2.14%, respectively. After branch girdling, the distribution ratio of Cd in root for CK-G group increased by 11.80% (*P* < 0.05), whereas those in stem, leaves, and bolls decreased by 28.29% (*P* < 0.05), 12.89% (*P* < 0.05), and 15.27% (*P* < 0.05), respectively, compared with CK. Compared with CK-G group, the distribution ratios of Cd in root, stem, and bolls for T1-G group increased by 3.98%, 1.06%, and 7.11%, respectively, whereas that in leaves decreased by 17.56% (*P* < 0.05); the distribution ratios of Cd in root, stem, and bolls for T2-G group increased by 2.78%, 14.39% (*P* < 0.05), and 5.69%, respectively, whereas that in leaves decreased by 17.80% (*P* < 0.05).

**Table 1:** Distribution ratios and transport indexes of Cd in cotton organs. Means in each row or column followed by the same lowercase letters are not significantly different (*P* < 0.05) by Duncan’s multiple range test. Data are means ± SD (n = 3).

	PTI	STI		DR			
	Root-stem	Stem-leaves	Stem-bolls	Root	Stem	Leaves	Bolls
CK	0.17 ± 0.01b	2.16 ± 0.11c	0.94 ± 0.05d	59.30 ± 2.97bc	9.94 ± 0.50c	21.43 ± 1.07bc	9.34 ± 0.47ab
T1	0.18 ± 0.01b	2.05 ± 0.10 cd	0.84 ± 0.04e	58.38 ± 2.92c	10.70 ± 0.53b	21.95 ± 1.10b	8.97 ± 0.45abc
T2	0.22 ± 0.01a	2.07 ± 0.10 cd	0.82 ± 0.04e	54.02 ± 2.70c	11.82 ± 0.59a	24.44 ± 1.22a	9.72 ± 0.49a
CK-J	0.11 ± 0.01 cd	2.95 ± 0.15a.	1.32 ± 0.07a	64.30 ± 3.21ab	6.77 ± 0.34e	19.96 ± 1.00 cd	8.97 ± 0.45abc
T1-J	0.10 ± 0.01d	2.25 ± 0.11c	1.30 ± 0.06a	67.88 ± 3.39a	7.06 ± 0.35e	15.90 ± 0.80e	9.17 ± 0.46abc
T2-J	0.11 ± 0.01 cd	2.23 ± 0.11c	1.18 ± 0.06b	67.20 ± 3.36a	7.44 ± 0.37de	16.58 ± 0.83e	8.78 ± 0.44bc
CK-G	0.11 ± 0.01 cd	2.62 ± 0.13b	1.11 ± 0.06bc	66.30 ± 3.32a	7.13 ± 0.36e	18.66 ± 0.93d	7.91 ± 0.40d
T1-G	0.10 ± 0.01d	2.14 ± 0.11c	1.18 ± 0.06b	68.94 ± 3.45a	7.20 ± 0.36e	15.39 ± 0.77e	8.47 ± 0.42 cd
T2-G	0.12 ± 0.01c	1.88 ± 0.09d	1.03 ± 0.05 cd	68.15 ± 3.41a	8.15 ± 0.41d	15.34 ± 0.77e	8.36 ± 0.42 cd

As shown in Table 1, compared with CK, the transport indexes of Cd from root to stem for T1 and T2 groups increased by 9.38% and 30.59% (*P* < 0.05), respectively, whereas those from stem to leaves for T1 and T2 groups decreased by 4.86% and 4.10%, respectively; in addition, the transport indexes of Cd from stem to bolls decreased by 10.82% (*P* < 0.05) and 12.45% (*P* < 0.05), respectively. Compared with CK-J group, the transport indexes of Cd from root to stem, from stem to leaves, and from stem to bolls for T1-J group decreased by 1.32%, 23.52% (*P* < 0.05), and 1.93%, respectively, and those from root to stem and from stem to bolls for T2-J group decreased by 24.36% (*P* < 0.05) and 10.89% (*P* < 0.05), respectively. Compared with CK-G group, the transport indexes of Cd from root to stem and from stem to leaves for T1-G group decreased by 2.81% and 18.42% (*P* < 0.05), respectively, whereas that from stem to bolls increased by 5.98%; the transport index of Cd from root to stem for T2-G group increased by 11.29%, whereas those from stem to leaves and from stem to bolls decreased by 28.14% (*P* < 0.05) and 7.61%, respectively.

### Effect of girdling and modifiers on chlorophyll pigment in cotton leaves

**Figure 2:**
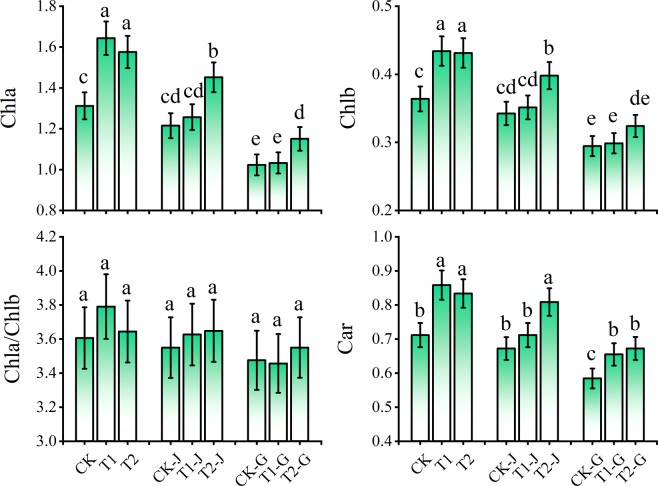
The relative changes of photosynthetic pigment contents in cotton leaves under different treatments. Means in each group followed by the same lowercase letters are not significantly different (*P* < 0.05) by Duncan’s multiple range test. Data are means ± SD (n = 3).

For the groups without girdling, the application of modifiers increased the *Chla*, *Chlb*, and *Car* concentrations of cotton leaves (Fig. 2). Compared with CK, the *Chla*, *Chlb*, and *Car* concentrations and the *Chla*/*b* ratio for T1 group increased by 25.23% (*P* < 0.05), 19.28% (*P* < 0.05), 5.11%, and 20.67% (*P* < 0.05), respectively, and those for T2 group increased by 20.10% (*P* < 0.05), 18.54% (*P* < 0.05), 1.05%, and 17.17% (*P* < 0.05), respectively. The stem girdling and branch girdling decreased the *Chla*, *Chlb*, and *Car* concentrations and the *Chla*/*b* ratio of leaves, whereas the application of modifiers alleviated the damage to photosynthetic pigments. After stem girdling, the *Chla*, *Chlb*, and *Car* concentrations and the *Chla*/*b* ratio for CK-J group decreased by 7.37%, 5.89%, 1.58%, and 5.53%, respectively, compared with CK. Compared with CK-J group, the *Chla*, *Chlb*, and *Car* concentrations and the *Chla*/*b* ratio for T1-J group increased by 3.41%, 2.60%, 2.17%, and 5.89%, respectively, and those for T2-J group increased by 19.46% (*P* < 0.05), 16.22% (*P* < 0.05), 2.78%, and 20.33% (*P* < 0.05), respectively. After branch girdling, the *Chla*, *Chlb*, and *Car* concentrations and the *Chla*/*b* ratio for CK-G group decreased by 21.99% (*P* < 0.05), 19.05% (*P* < 0.05), 3.62%, and 17.87% (*P* < 0.05), respectively, compared with CK. Compared with CK-G group, the *Chla*, *Chlb*, and *Car* concentrations for T1-G group increased by 0.87%, 1.42%, and 12.11% (*P* < 0.05), respectively, and the *Chla*, *Chlb*, and *Car* concentrations and the *Chla*/*b* ratio for T2-G group increased by 12.41% (*P* < 0.05), 10.05% (*P* < 0.05), 2.13%, and 15.09% (*P* < 0.05), respectively.

### Effect of girdling and modifiers on photosynthesis parameters of cotton leaves

**Figure 3:**
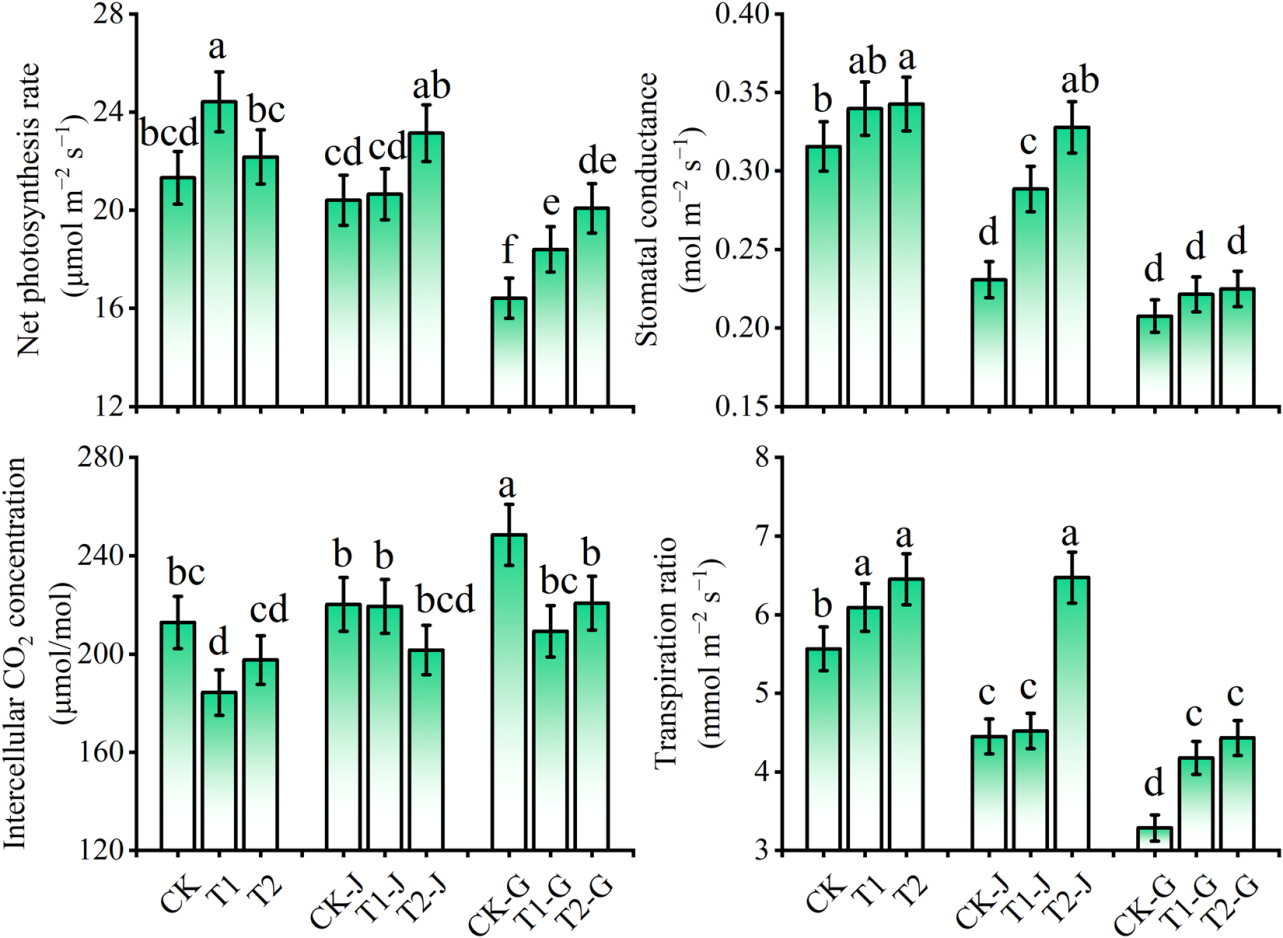
The relative changes of photosynthesis parameters of cotton leaves under different treatments. Means in each group followed by the same lowercase letters are not significantly different (*P* < 0.05) by Duncan’s multiple range test. Data are means ± SD (n = 3).

For the groups without girdling, the application of modifiers increased the net photosynthesis rate (*Pn*) of cotton leaves (Fig. 3). Compared with CK, the *Pn* for T1 and T2 groups increased by 14.51% (*P* < 0.05) and 3.98%, respectively. The stem girdling and branch girdling decreased the leaf *Pn*. The *Pn* for CK-J and CK-G groups decreased by 4.30% and 23.02% (*P* < 0.05), respectively, compared with CK. The application of modifiers alleviated the decreases of *Pn* caused by girdling. Compared with CK-J group, the *Pn* for T1-J and T2-J groups increased by 1.19% and 13.39% (*P* < 0.05), respectively. Compared with CK-G group, the *Pn* for T1-G and T2-G groups increased by 12.14% (*P* < 0.05) and 22.34% (*P* < 0.05), respectively.

Modifiers application and girdling treatments significantly affected the stomatal conductance (*Gs*), intercellular CO_2_ concentration (*Ci*), and transpiration rate (*Tr*) of cotton leaves. For the groups without girdling, the application of modifiers increased the *Gs* and *Tr* but decreased the *Ci* (Fig. 3). After stem girdling, the *Gs* and *Tr* for CK-J group decreased by 26.86% (*P* < 0.05) and 20.02% (*P* < 0.05), respectively, whereas the *Ci* increased by 3.43% compared with CK; the *Gs* and *Tr* for CK-G group decreased by 34.21% (*P* < 0.05) and 40.96% (*P* < 0.05), respectively, whereas the *Ci* increased by 16.73% (*P* < 0.05) compared with CK. The application of modifiers also significantly changed the *Gs*, *Ci* and *Tr* of cotton leaves. Among them, compared with CK-J group, the *Gs* for T1-J and T2-J groups increased by 25.03% (*P* < 0.05) and 42.03% (*P* < 0.05), respectively, and the *Tr* for T1-J and T2-J groups increased by 1.53% and 45.44% (*P* < 0.05), respectively; whereas the *Ci* for T1-J and T2-J groups decreased by 0.37% and 8.45%, respectively. Compared with CK-G group, the *Gs* for T1-G and T2-G groups increased by 6.71% and 8.38%, respectively, and the *Tr* for T1-G and T2-G groups increased by 27.13% (*P* < 0.05) and 34.90% (*P* < 0.05), respectively; whereas the *Ci* for T1-G and T2-G groups decreased by 15.80% (*P* < 0.05) and 11.20% (*P* < 0.05), respectively.

### Effect of modifiers on microstructure of cotton leaf tissue

**Figure 4:**
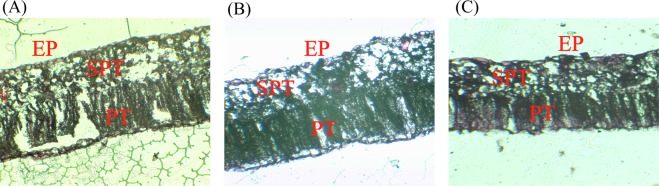
Effect of Modifiers on Microstructure of Cotton Leaves (eyepiece × objective lens: 10 × 40). EP: Epidermis; PT: Palisade tissue; SPT: Sponge tissue. (**A**) CK, (**B**) T1 group, (**C**) T2 group.

The observation of the paraffin sections of leaves in CK, T1, and T2 groups showed that leaves are composed of epidermis, palisade tissue, and sponge tissue (Fig. 4). For the control group (CK), leaf tissues were irregularly arranged; some cells contracted, shortened, and even curved, and the gap between cells increased. Compared with T1 and T2 groups, the number of sponge tissue cells for CK was decreased, the arrangement was loose, the intercellular spaces were increased, and the cells were decreased. For T1 and T2 groups, the palisade tissue cells of cotton leaves were long columnar; the cells were arranged neatly and tightly, and there were more chloroplasts compared with CK. The rounded sponge tissue cells were arranged closely.

### Redundancy analysis of cotton physiological characteristics

**Figure 5:**
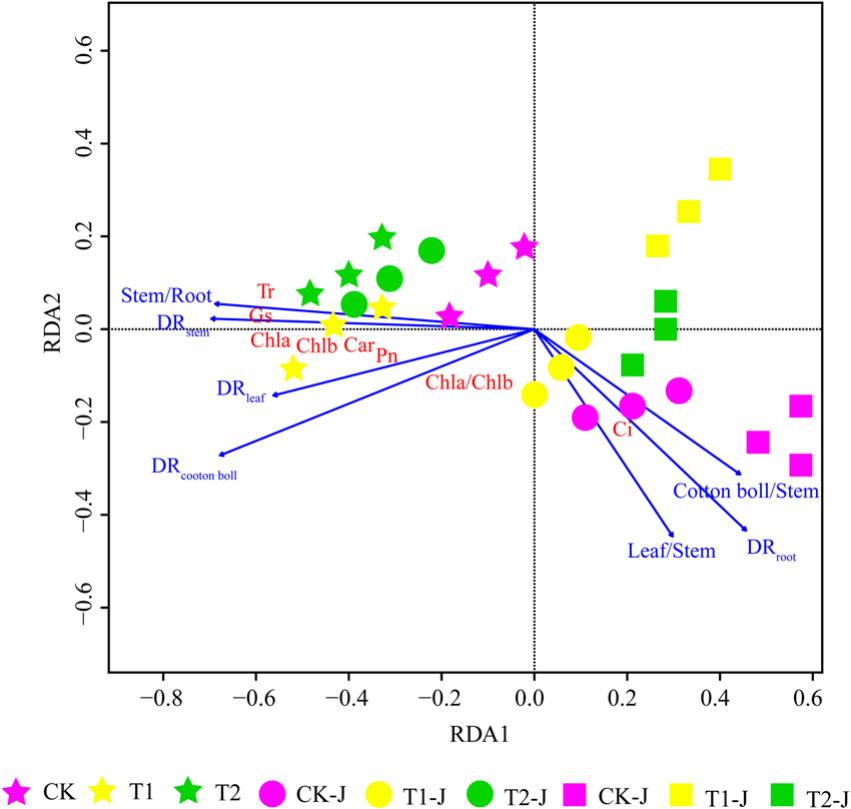
RDA analysis of transport and distribution of Cd and photosynthetic parameters of cotton.

The effects of modifiers on Cd accumulation and leaf photosynthetic parameters for different groups were analyzed by redundancy analysis (RDA). As shown in Fig. 5, PTI_root-stem_, DR_stem_, DR_leaves_, and DR_bolls_ were clearly distributed on the left side of the RDA1 axis. PTI_root-stem_ and DR_stem_ were closely related to the *Tr* and *Gs*, whereas DR_leaves_ was closely related to the *Pn*. STI_stem-leaves_, STI_stem-bolls_, and DR_root_ were clearly distributed on the right side of the RDA1 axis. The *Ci* was closely related to DR_root_. and PTI_root-stem_ and DR_stem_ were closely related to T1 and T2 groups. After stem girdling, T1-J group was closely related to the *Chla*/*b* and *Ci*, and T2-J group was closely related to the *Chla*, *Chlb*, *Car*, *Tr*, and *Gs*. After branch girdling, T2-G group was closely related to the *Ci*.

## Discussion

Most heavy metals such as Pb, Cr, and Cu are absorbed and stored in crop root, while Cd, Ni, and Zn, etc. are easily transported to the upper part of crops^16–18^. At present, many plants have been studied in terms of the Cd distribution and accumulation^8,18^, and the ability of xylem to transport Cd into shoots has been considered as a major determinant of Cd accumulation in the shoots of many plants^7^. In addition, the absorption of Cd by peanut and wheat also showed that Cd was mobile in phloem tissue^19^. According to the previous results, we studied the distribution and transport of Cd in cotton organs by girdling, and found that the mobile of Cd in different organs of cotton was obvious. For the control group (CK), the root had the highest Cd accumulation, followed by leaves, stem, and bolls (Fig. 1). From the distribution ratio, we found that stem girdling significantly reduced the accumulation of Cd in stem, and branch girdling not only significantly reduced the accumulation of Cd in stem, but also reduced that in leaves and bolls (Table 2). It indicated that Cd was of high mobility in both xylem and phloem, and stem may play an important role in transport of Cd for the two pathways. This is same as the research result by Reid *et al*.^19^: Cd was transported through xylem and phloem.

**Table 2:** Basic properties of modifiers used.

Modifiers	Properties
Polyacrylate compound modifier	A compound modifier composed of polypropylene and iron sulfate. A colorless liquid that features in surface adsorption and co-precipitation for metals
Organic polymer compound modifier	A compound modifier mainly composed of polyacrylamide and manganese sulfate. A colorless liquid that features in surface adsorption, surface complexation, etc.

The accumulation and transport of Cd in crops have been regulated through the application of exogenous materials in previous studies. For example, Huang *et al*.^20^ reported that the application of calcium can reduced the Cd accumulation in soybean and wheat roots. Guiwei *et al*.^21^ found that the application of polyacrylate polymers reduced the bioavailable Cd, which could be employed to enhance productivity of crops in Cd-contaminated soils. In this study, the modifiers used are mainly polymer materials. The carboxyl and amide groups of polyacrylamides can form coordination bonds with metal ions on the surface of soil particles to form complexes^22^. From transport indexes, we found that the polyacrylate compound modifier significantly reduced the transport of Cd from stem to bolls, and the organic polymer compound modifier significantly reduced the transport of Cd from root to stem and from stem to bolls (Table 2), indicating that polyacrylate compound modifier mainly reduced the mobility of Cd in cotton, and organic polymer compound modifier mainly reduced the absorption of Cd by cotton. Moreover, it was found that the application of polyacrylate compound modifier reduced the transport of Cd from stem to leaves after stem girdling and branch girdling, and the application of organic polymer compound modifier reduced the transport of Cd from stem to leaves after stem girdling and the transport of Cd from stem to bolls after branch girdling (Table 2), indicating that modifiers could be transported to leaves and bolls after phloem girdling. According to RDA analysis, without girdling, the application of polyacrylate compound modifier could significantly change the distribution and transport of Cd in cotton organs compared with CK, whereas the application of organic polymer compound modifier could significantly change the distribution and transport of Cd in cotton organs compared with CK-J and CK-G groups after girdling.

Many studies have found that phloem girdling reduces the net photosynthetic rate (*Pn*) of plant leaves^23^. Sala *et al*.^24^ showed that phloem girdling influenced the rate of photosynthetic assimilation, and Poirier-Pocovi *et al*.^25^ showed that phloem girdling reduced the photosynthesis rate of leaves. Similar findings were also shown in this study. After girdling, the *Pn, Gs*, and *Tr* all decreased, and only *Ci* increased, indicating that the decrease of photosynthetic rate was mainly caused by non-stomatal factors^26^. This is consistent with the conclusion of Zhou *et al*.^27^. Without girdling, the applications of the two modifiers significantly decreased the inhibition from Cd on *Pn* of cotton leaves. For groups with girdling, only T2-J group significantly decreased the inhibition on *Pn*, but there was significant difference in the decreases of *Pn* among the groups treated with modifiers. The results indicated that the modifiers could alleviate the photosynthetic damage caused by girdling. After stem girdling, the *Pn* for the groups treated with organic polymer compound modifier were higher than that of CK (Fig. 3), which indicated that the application of organic polymer compound modifier after stem girdling had a positive effect on *Pn*. This might be due to the application of organic polymer compound modifier decreased intercellular CO_2_ concentration, but increased stomatal conductance and transpiration rate (Fig. 3).

Consistent with the research results of Tang *et al*.^28^, the pigment chlorophyll of cotton leaves was significantly decreased by girdling, which helps to determine the compensatory photosynthesis by modifiers (Fig. 2). The decreases of *Chla*, *Chlb*, and *Car* concentrations for the groups treated with branch girdling was greater than those for the groups treated with stem girdling, and the changes of photosynthetic rates also showed the same phenomenon. It indicated that the damage caused by branch girdling was more serious. This may be due to the accumulation of carbohydrates in the upper part above the cut after girdling. Photosynthates could not be transported to the root, thus photosynthesis was inhibited. At this time, the plant might avoid absorbing too much light by reducing the content of photosynthetic pigments. This reaction is the feedback regulation according to the source-sink relation^25^. The application of modifiers improved the photosynthesis of leaves because the chlorophyll content was significantly increased, and the effect of the application of organic polymer compound modifier after stem girdling was more obvious. Moreover, *Chla* and *Chlb* play different roles in photosynthesis^29^. The ratios of *Chla*/*b* in cotton leaves decreased significantly after girdling, but they were increased after the application of modifiers, indicating that after girdling, the application of modifiers increased the number of molecules involved in photochemical reaction, which led to the improvement of photosynthesis^29^. According to the RDA analysis, the polyacrylate compound modifier has a greater effect on the protection of pigment chlorophyll of cotton without girdling than the organic polymer compound modifier, whereas the organic polymer compound modifier has a greater effect on that of cotton with girdling. In addition, Liu *et al*.^30^ found that Cd distributed in leaf epidermis, spongy tissue, and palisade tissue of crops in Cd-contaminated soils. By observing the microstructure of leaf tissues, we found that Cd did not damage the epidermis cells but the palisade tissue cells in mesophyll and sponge tissue cells, which would affect chloroplasts. Because Cd was absorbed by cotton root and transported from root to shoots, there was no direct contact with epidermis cells. By applying the modifiers, the densities of palisade tissue and sponge tissue were adjusted, which promoted the development of mesophyll cells and chloroplasts and increased the chlorophyll content. Thus, the photosynthesis of cotton leaves was improved.

## Conclusions

This study proved that Cd is of high mobility in both xylem and phloem through girdling. The transport pathways of polyacrylate compound modifier and organic polymer compound modifier in cotton were the same as that of Cd. Modifies could still be transported to the shoots after phloem girdling, but the effect of modifiers on alleviating Cd stress was not greater than that without girdling. Moreover, on the one hand, the application of the two modifiers could stabilize Cd in cotton root and reduce the absorption of Cd; on the other hand, it could alleviate the inhibition of photosynthesis caused by phloem girdling.

## Materials and Methods

### Test site condition

Test soil (Calcaric Fluvisol) was obtained from the Test Station of Agricultural College, Shihezi University in Shihezi City, Xinjiang Province, China (86°03′E, 45°19′N). The soil pH was 7.76, soil cation-exchange capacity (CEC) was 16.25 coml kg^−1^, total nitrogen content was 0.89 g kg^−1^, organic matter content was 13.25 g kg^−1^, alkali-hydrolyzable nitrogen content was 60 mg kg ^−1^, available phosphorus content was 20 mg kg ^−1^, and available potassium content was 250 mg kg^−1^^31^.

### Test program

Two-year continuous remediation of Cd-contaminated soil using polymer modifiers was conducted in 2017 and 2018. On April 5th, 2017, to maintain the original soil profile, soils were packed into plastic barrels (length × width × height = 30 cm × 30 cm × 80 cm), and then barrels were buried back into the field. Based on previous studies^14^ and pre-test results, cadmium chloride (CdCl_2_•2.5H_2_O) solution was added into the barrels and mixed fully with the plough layer. The Cd content in the plough layer reached approximately 40 mg kg^−1^ after three weeks. On April 26th, urea (345 kg hm^−2^) and compound fertilizer (17-17-17) (795 kg hm^−2^) was applied.

A total of nine groups were set in a randomized block design, and two treatments were employed, including: (1) the application of modifiers (non-modifier, polyacrylate compound modifier, and organic polymer compound modifier (Table 3)); and (2) phloem girdling (non-girdling, stem gridling, and branch girdling) (Table 2). Each group had three repetitions. On April 30th, two modifiers (8.48 kg hm^−2^) were diluted with water and applied through drip irrigation. Cotton (Xinluzao 60) was sown on May 2th. After emergence, six seedlings were retained in each barrel. The first irrigation was conducted on June 14th. The irrigation cycle was 3 days during the growth period, and the irrigation volume was 4,500 m^3^ hm^−2^. No fertilizers and modifiers were applied at later stages. Cotton was harvested on September 5, 2017. After that, the stalks were pulled out and the field was plowed.

**Table 3:** Test design.

Group	Phloem girdling	Modifier application
CK	No girdling	/
T1	No girdling	Polyacrylate compound modifier
T2	No girdling	Organic polymer compound modifier
CK-J	Stem gridling	/
T1-J	Stem gridling	Polyacrylate compound modifier
T2-J	Stem gridling	Organic polymer compound modifier
CK-G	Branch girdling	/
T1-G	Branch girdling	Polyacrylate compound modifier
T2-G	Branch girdling	Organic polymer compound modifier

On April 5th, 2018, the Cd content in plough layer reached 32.44 mg kg^−1^ in the soil without modifiers, 31.57 mg kg^−1^ in the soil with polyacrylate compound modifier, and 31.22 mg kg^−1^ in the soil with organic polymer compound modifier. In 2018, no cadmium was applied into the soils, and the amounts of modifiers applied (8.48 kg hm^−2^) and the other managements were the same as those in 2017.

To evaluate the transport pathway of Cd (phloem or xylem), and analyze the effects of modifiers on Cd content and regulating photosynthesis in cotton, phloem girdling was employed to inhibit the transport of Cd and modifiers through phloem in 2018. Phloem girdling was conducted at the cotton flowering boll-setting stage (the 10th day after manual topping). Girdling sites of stem girdling and branch girdling were located at the main stem of cotton (3–5 cm from the ground) and the top fruit branch, respectively. The width of the cut was 1 cm, and the depth was up to the xylem. Ten days after girdling, functional leaves from each barrel were randomly selected to determine photosynthetic parameters, and three cotton plants were randomly selected from each barrel to determine Cd content.

### Experimental determination

#### Measurement of Cd content

Root, stem, leaves, and cotton bolls of cotton plants were separated, and then they were dried at 105 °C for 0.5 h and dried to constant at 80 °C. Samples of each organ (0.5 g) were acid-digested using sulfuric and nitric acid (1:5, v/v) at 60 °C for 24 h, and treated with HNO_3_/HClO_4_ (5/1, v/v). A Hitachi Z2000 graphite atomic absorption spectrophotometer (PinAAcle900T, PerkinElmer, USA) was used for the determination of Cd content^31^.

#### Chlorophyll pigment measurement

Fresh leaves (20 mg) from each group were cut into small pieces. Photosynthetic pigments were extracted in 80% (v/v) acetone, and centrifuged. Pellets were used to re-extract the pigments until they became colorless. The absorbance was determined at 663.2, 646.5, and 470 nm spectrophotometrically. The concentrations of chlorophyll (*Chla* and *Chlb*) and carotenoids (*Car*) were calculated according the method of Lichtenthaler^32^.

#### Photosynthesis parameters

Net photosynthetic rate (*Pn*), stomatal conductance (*Gs*), transpiration rate (*Tr*), and intracellular CO_2_ concentration (*Ci*) were determined with a portable photosynthesis system LI-6400 (LI-COR, Lincoln, USA) according to Liu *et al*.^33^.

#### Anatomical type of cotton leaves

In the flowering and boll-setting stage, the leaves at the same position of cotton plants in each group were collected and wrapped up using wet gauze. After that, they were put in ice box and taken back to the laboratory. Leaves were washed with distilled water, and then, they were blotted with absorbent paper. A small piece (about 1 cm × 0.5 cm) was cut with a blade and put into the FAA fixing solution (formaldehyde: 5 mL, glacial acetic acid: 5 mL, and 70% alcohol: 90 m L) for making paraffin sections. Paraffin sectioning^34^ was conducted using hematoxylin and Canadian neutral gum. The thickness of the slice was 6 to 8 μm, and the slices were photographed with a microscope (eyepiece × objective lens: 10 × 40) (DMB: Motic Digital Microscope).

### Statistical analysis

The data were processed using Excel 2016 (Microsoft, USA), and one-way analysis of variance (ANOVA) was performed using SPSS 23.0 (SPSS Inc., Chicago, IL, USA). Multiple comparisons between different groups were conducted using Duncan’s new multiple range method (significance level: α = 0.05). Charts were drawn using Origin 8.0 (Origin Lab, Massachusetts, USA).

Calculation formulas in data processing:

1 $${\rm{DR}}({\rm{Distribution}}\,{\rm{ratio}})={\rm{Cd}}\,{\rm{uptake}}\,{\rm{in}}\,{\rm{root}}({\rm{stem}},\,{\rm{leaves}})/{\rm{total}}\,{\rm{Cd}}\,{\rm{uptake}}\,{\rm{of}}\,{\rm{the}}\,{\rm{plant}}$$

2 $${\rm{P}}{\rm{T}}{\rm{I}}({\rm{P}}{\rm{r}}{\rm{i}}{\rm{m}}{\rm{a}}{\rm{r}}{\rm{y}}\,{\rm{t}}{\rm{r}}{\rm{a}}{\rm{n}}{\rm{s}}{\rm{p}}{\rm{o}}{\rm{r}}{\rm{t}}\,{\rm{i}}{\rm{n}}{\rm{d}}{\rm{e}}{\rm{x}})={\rm{C}}{\rm{d}}\,{\rm{c}}{\rm{o}}{\rm{n}}{\rm{t}}{\rm{e}}{\rm{n}}{\rm{t}}\,{\rm{i}}{\rm{n}}\,{\rm{s}}{\rm{t}}{\rm{e}}{\rm{m}}/{\rm{C}}{\rm{d}}\,{\rm{c}}{\rm{o}}{\rm{n}}{\rm{t}}{\rm{e}}{\rm{n}}{\rm{t}}\,{\rm{i}}{\rm{n}}\,{\rm{r}}{\rm{o}}{\rm{o}}{\rm{t}}$$

3 $${\rm{S}}{\rm{T}}{\rm{I}}({\rm{S}}{\rm{e}}{\rm{c}}{\rm{o}}{\rm{n}}{\rm{d}}{\rm{a}}{\rm{r}}{\rm{y}}\,{\rm{t}}{\rm{r}}{\rm{a}}{\rm{n}}{\rm{s}}{\rm{p}}{\rm{o}}{\rm{r}}{\rm{t}}\,{\rm{i}}{\rm{n}}{\rm{d}}{\rm{e}}{\rm{x}})={\rm{C}}{\rm{d}}\,{\rm{c}}{\rm{o}}{\rm{n}}{\rm{t}}{\rm{e}}{\rm{n}}{\rm{t}}\,{\rm{i}}{\rm{n}}\,{\rm{c}}{\rm{o}}{\rm{t}}{\rm{t}}{\rm{o}}{\rm{n}}\,{\rm{b}}{\rm{o}}{\rm{l}}{\rm{l}}{\rm{s}}({\rm{l}}{\rm{e}}{\rm{a}}{\rm{v}}{\rm{e}}{\rm{s}})/{\rm{C}}{\rm{d}}\,{\rm{c}}{\rm{o}}{\rm{n}}{\rm{t}}{\rm{e}}{\rm{n}}{\rm{t}}\,{\rm{i}}{\rm{n}}\,{\rm{s}}{\rm{t}}{\rm{e}}{\rm{m}}$$
